# Arzanol, a Potent mPGES-1 Inhibitor: Novel Anti-Inflammatory Agent

**DOI:** 10.1155/2013/986429

**Published:** 2013-10-01

**Authors:** Pankaj S. Kothavade, Dnyaneshwar M. Nagmoti, Vipin D. Bulani, Archana R. Juvekar

**Affiliations:** Department of Pharmaceutical Sciences and Technology, Institute of Chemical Technology, Nathalal Parekh Marg, Matunga, Mumbai 400 019, India

## Abstract

Arzanol is a novel phloroglucinol **α**-pyrone, isolated from a Mediterranean plant *Helichrysum italicum* (Roth) Don ssp. *microphyllum* which belongs to the family Asteraceae. Arzanol has been reported to possess a variety of pharmacological activities. However, anti-inflammatory, anti-HIV, and antioxidant activities have been studied in some detail. Arzanol has been reported to inhibit inflammatory transcription factor NF**κ**B activation, HIV replication in T cells, releases of IL-1**β**, IL-6, IL-8, and TNF-**α**, and biosynthesis of PGE_2_ by potentially inhibiting mPGES-1 enzyme. Diversity of mechanisms of actions of arzanol may be useful in treatment of disease involving these inflammatory mediators such as autoimmune diseases and cancer. This review presents comprehensive information on the chemistry, structure-activity relationship, and pharmacological activities of arzanol. In addition this review discusses recent developments and the scope for future research in these aspects.

## 1. Introduction

 Inflammation is the reaction of living tissue to injury, in which a series of changes of the terminal vascular bed, blood, and connective tissue tends to eliminate the injurious agent and to repair the damaged tissue [1]. Inflammatory process involves evidence of prostaglandins, leukotrienes, histamine, bradykinin, platelet-activating factor (PAF), and interleukins (IL) [2]. Eicosanoids, namely, prostaglandins and leukotrienes, are key substances involved in inflammatory reactions; hence, these eicosanoids inhibition is major target for anti-inflammatory agents. Nonselective cyclooxygenase (COX) inhibitors (Diclofenac, Indomethacin), selective COX-2 inhibitors (Celecoxib, Etoricoxib), phospholipase A_2_ (PLA_2_) inhibitors (Corticosteroids) and newer therapies such as antitumour necrosis factor (TNF)-*α* therapy (Etanercept, Infliximab, and Adalimumab), and anti-CD20 therapy (Rituximab) are often used to inhibit the underlying immune process. However, long-term clinical use of these agents is associated with risk of adverse effects, as they have been linked with gastrointestinal toxicity and an increased risk of adverse cardiovascular events. In recent years, researchers are involved in finding new categories of the development of other enzymatic targets within the arachidonic acid pathway, including the PGE_2_ synthase (PGES) to overcome the side effect of existing compounds. Several compounds like MF-63, NS-398, MK-866, and Triclosan are microsomal PGE_2_ synthase (mPGES) inhibitors, which were assayed for *in vitro* studies, but some of them have shown poor bioavailability and hepatotoxicity [3]. 

*Helichrysum italicum* (Roth) Don ssp. *microphyllum* is mediterranean plant belongs to the Asteraceae family. This plant is widespread in the areas of stony, arid, sandy mediterranean region along the east coast and on the islands of the Adriatic Sea [4]. *H. italicum *has been reported to possess anti-inflammatory, antioxidant [5], antiviral [6], antimicrobial [7], and antifungal activities [8]. An array of phytochemical constituents, namely, *α*-amyrin, uvaol [9], beta-diketones [10], kaempferol-3-glucoside, naringenin-glycoside [11], gnaphaliin, pinocembrin, tiliroside [12], eudesm-5-en-11-ol [13],* iso*-italicene epoxide, *β*-costol, (*Z*)-*α*-*trans*-bergamotol [14], and phloroglucinol *α*-pyrone arzanol [15], were reported in* H. italicum*. 

Appendino et al. [15] started systematic study of phloroglucinol *α*-pyrone class of compounds which possessed anti-inflammatory activity. They developed isolation techniques with high yield of compounds and screened them for anti-inflammatory activity. Arzanol, a prenylated heterodimeric phloroglucinyl pyrone, was firstly identified in early 2007 and detected as a major anti-inflammatory compound. It inhibits NF*κ*B (nuclear factor kappa B) activation, release of proinflammatory mediators like interleukins (IL), TNF-*α*, prostaglandin E_2_ (PGE_2_) [15], and inducible microsomal PGE_2_ synthase-1 (mPGES-1) [16]. Arzanol also inhibits HIV-1 replication in T cells; hence it has anti-HIV property [15]. Antioxidant and cytotoxic action of arzanol were studied by [17]. Hence, present review will focus on the isolation, chemistry, and pharmacology of the arzanol.

## 2. Isolation and Identification of Arzanol

**Figure 1:**
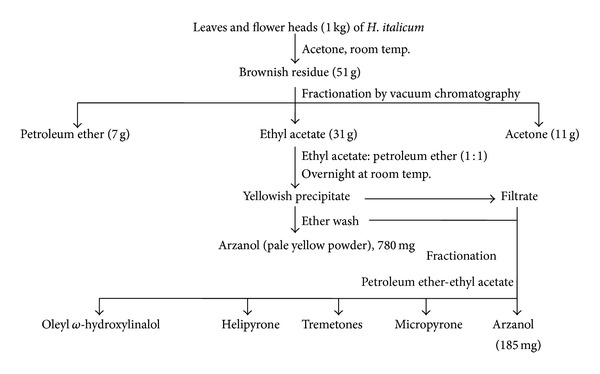
Schematic representation of isolation of phytochemical constituents of *H. italicum* subsp.* microphyllum*. (Adapted and modified from Appendino et al. [15].)

The *H. italicum*, a best known medicinal plant from the Mediterranean area, was used as an anti-inflammatory and anti-infective plant in Greek-Roman system of medicine. Appendino et al. [15] developed an extraction and isolation procedure of arzanol from the acetone extract of aerial parts, mainly leaves and flower heads of *H. italicum*. The acetone extract showed potent NF-*κ*B-inhibitory activity; in an attempt to identify the active compounds, the acetone extract was separated by solid-phase extraction into three fractions (petroleum ether-ethyl acetate, and acetone). They succeeded in isolating a pale yellow compound with powerful NF-*κ*B inhibiting activity (IC_50_ = 5 **μ**g/mL), superior to that of all the other products obtained by bioassay-directed fractionation of the mother liquors by gravity column chromatography. Approximately 780 mg of arzanol was isolated from the extracts representing 1 kg dried aerial parts, mainly leaves and flower heads of *H. italicum*, as shown in Figure 1 and was identified and characterized by spectroscopic methods [15–18].

## 3. Chemistry of Arzanol

**Figure 2:**
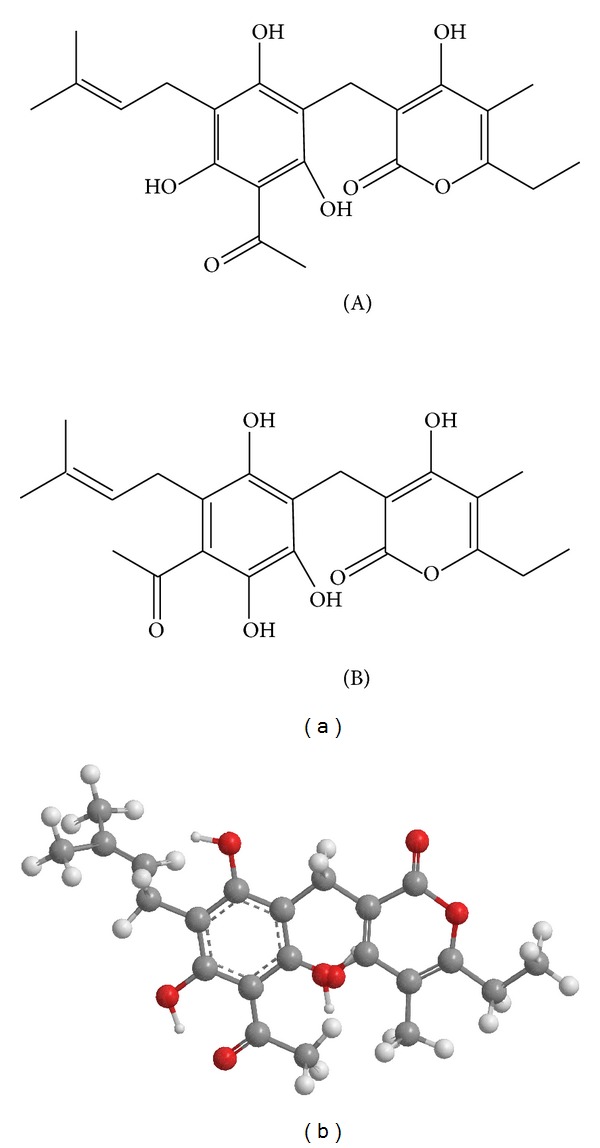
(a) Chemical structure of arzanol (A) and homoaranol (B), (b) 3D structure of arzanol: 3-(3-acetyl-2,4,6-trihydroxy-5-(3-methylbut-2-en-1-yl)benzyl)-6-ethyl-4-hydroxy-5-methyl-2H-pyran-2-one.

 Arzanol belongs to phloroglucinol *α*-pyrone category. Structure of arzanol is identical with homoarenol, (Figure 2(a)) which was isolated from *H. arenarium *L. Moench species [19]. Appendino et al. [15] derived the name “arzanol” on the basis of plant *H. italicum *collected from Sardinian village—Arzana.

 Arzanol is a prenylated heterodimeric phloroglucinyl *α*-pyrone chemically known as 3-(3-acetyl-2,4,6-trihydroxy-5-(3-methylbut-2-en-1-yl)benzyl)-6-ethyl-4-hydroxy-5-methyl-2H-pyran-2-one (Figure 2(b)).

### 3.1. Synthesis of Arzanol (Figure 3) [20, 21]

#### 3.1.1. Step I: Formation of Prenylated Acylphloroglucinol

 Phloroacetophenone (i) reacted with *tert*-butyldimethylsilyl chloride (TBDMSCl) for the protection of nonchelated hydroxyls of phloroacetophenone. In presence of prenyl alcohol, tetraphenylporphyrin (TPP) and toluene phloroacetophenone are converted into prenylated acylphloroglucinol (ii).

#### 3.1.2. Step II: Formation of Reactive Methylene-2,4 Dioxypyrone

 Acylation of the dianion of ethyl 3-oxopentanoate (iv) with ethyl acetoacetate followed by cyclodehydration lead to formation of *α*-pyrone (vi). Compound (vi) which reacted with paraformaldehyde gives reactive methylene-2,4 dioxypyrone.

#### 3.1.3. Step III: Desilylation of Prenylated Acylphloroglucinol and Formation of Arzanol

 Indirect deprotonation of the phloroacetophenone (iii) by fluoride-induced desilylation using TBAF (tetrabutylammonium fluoride) and mixture of compound (vi) and paraformaldehyde in a warm 40°C chloroform affords arzanol (vii) with yield of 60%.

### 3.2. Structure-Activity Relationship (SAR) of Arzanol

**Figure 4:**
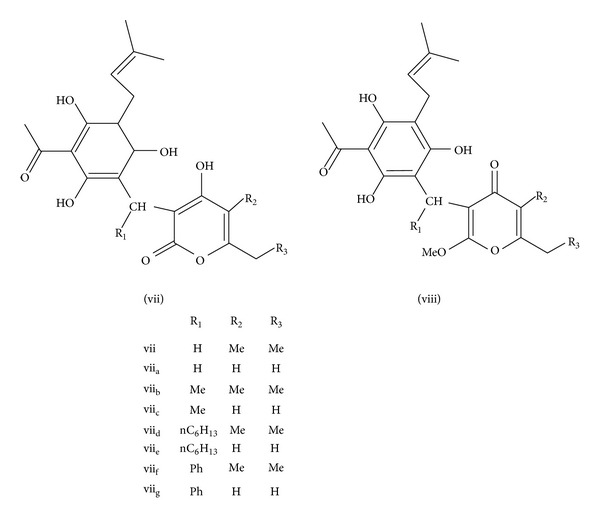
Structure-activity relationship of arzanol and its derivatives. (Adapted from Minassi et al. [21].)

Minassi et al. [21] focussed on the alkylidene linker and pyrone moiety to find out structure activity relationship of arzanol (Figure 4). The pharmacological activity of derivatives of arzanol was assayed using inhibitory potency towards mPGES-1 and 5-LOX.

- In a compound (vii_a_) replacement of CH_3_ to H at R_2_ and R_3_ position from pyrone ring does not affect the biological activity.
- Addition of methyl group at R_1_ on alkylidene linker (vii_b_ and vii_c_) slightly decreased the activity against mPGES-1 and 5-LOX.
- n-Hexyl residue attached to R_1_ (vii_d_ and vii_e_) to analyse the effect of elongated alkyl group leads to beneficial effect. Those compounds have higher mPGES-1 and 5-LOX inhibitory potency over vii_a_.
- The benzylidene derivatives (vii_f_ and vii_g_) showed an increased potency towards mPGES-1; hence lipophilic compound at alkylidene linker is beneficial for bioactivity.
- O-alkylation of pyrone moiety (viii) have only 5-LOX inhibitory activity and inactive for inhibition of mPGES-1 activity. It suggests that presence of acidic group is responsible for mPGES-1 activity of arzanol.

## 4. Pharmacological Role of Arzanol in Treatment of Inflammation

Acetophenone, *γ*-pyrone, and flavonoids have been isolated from the aerial parts of *H. italicum*. 4-Hydroxy-3-(3-methyl-2-butenyl) acetophenone reported inhibitory effect on cyclooxygenase and 5-lipoxygenase, while *γ*-pyrone glycoside acts as inhibitor of phospholipase A_2_. Flavonoids, namely, gnaphaliin, pinocembrin, and tiliroside, were screened for antioxidant and anti-inflammatory activity. Tilirosides were found to be potent anti-inflammatory flavonoids with respect to Phospholipase A_2_ inhibition [5, 12, 22]. Phloroglucinol *α*-pyrone (Arzanol), an isolated compound from acetone extract of *H. italicum *and have potent mPGES-1 inhibitor activity [16].

**Figure 5:**
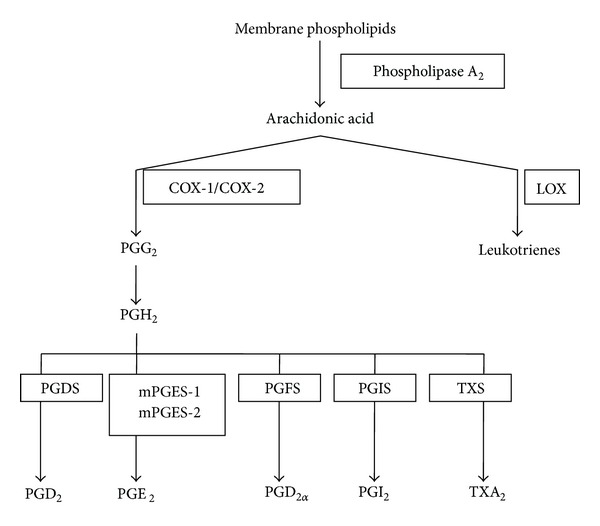
Biosynthesis of eicosanoid. (Adapted and modified from Dallaporta et al. [23].)

 Eicosanoid biosynthesis involves various enzymes such as phospholipase, cyclooxygenase (COX), lipoxygenase (LOX), and microsomal PGE_2_ synthase (mPGES-1/mPGES-2) enzymes (Figure 5). Phospholipase A_2_ activated by some chemical and mechanical stimuli results in release of arachidonic acid from membrane phospholipids. COX-1/COX-2 is responsible for conversion of arachidonic acid to PGG_2_ and further into PGH_2_. Prostanoids like PGD_2_, PGE_2_, PGF_2*α*_, PGI_2_, and TXA_2_ are synthesised from PGH_2_ in cell-specific manner by PGDS, mPGES-1/mPGES-2, PGFS, PGIS, and TXS enzymes, respectively. LOX is involved in synthesis of leukotrienes (LTA_4_, LTB_4_, LTC_4_, LTD_4_, and LTE_4_) [23–25]. Prostanoids, that is, PGI_2_, PGE_2_, and TXA_2_, are derived from COX enzyme_._ PGI_2_ derived from COX-2 plays a role in induction of vasodilatation, inhibition of platelet aggregation, vascular smooth muscle cell proliferation inhibition, and decreased cholesterol accumulation [26]. COX-1 derived TXA_2_ is responsible for vasoconstriction and platelet aggregation; this effect of TXA_2_ does not alter by the use of COX-2 inhibitors. Hence, imbalance between PGI_2_ and PGE_2_ is responsible for cardiovascular side effects of COX-2 inhibitors [23, 27].

 Prostaglandin E_2_ (PGE_2_) is a bioactive lipid synthesized by sequential action of cyclooxygenase, which specifically converts arachidonic acid into prostaglandin H_2_ (PGH_2_). A downstream signaling cascade by specific terminal membrane associated PGES-1 (mPGES-1), PGES-2 (mPGES-2) and cytosolic PGES (cPGES) have been identified and demonstrated to possess specific enzymatic activity mediating the conversion of PGH_2_ into PGE_2_ (Figure 5). mPGES-1 is a membrane bound, glutathione dependant enzymes involved in eicosanoids metabolism, induced by proinflammatory cytokines. It was proposed that mPGES-1 is functionally coupled to COX-2 and mediates the late phase of PGE_2_ production [28, 29]. Overexpression of COX-2, IL-1*β*, and tumor necrosis factor (TNF)-*α* causes rises in level of mPGES-1 which leads to production of PGE_2_ in inflammation [3, 29, 30]. PGE_2_ plays critical roles in wide range of biological processes, including inflammation, cancer, blood pressure regulation, pain sensation, febrile response, and reproduction [31]. The physiological roles of PGE_2_ are mediated in part through activation of key downstream signaling cascades via transmembrane EP receptors located on the cell surface. PGE_2_ receptors EP1, EP2, EP3, and EP4 are present in spinal neurons [pain response], ovarian cells [maturation of ovulation], organum vasculosum lamina terminalis (OVLT) at the midline of the preoptic area (POA) [Fever generation], and osteoclast [Bone resorption], respectively [32]. Receptor specific binding can activate diverse pathways that regulate cell proliferation, apoptosis, angiogenesis, inflammation, and immune surveillance. Hence,targeting to mPGES leads to decrease in PGE_2_ levels and help in reduction of cardiovascular side effects of the COX-2 inhibitors.

**Figure 6:**
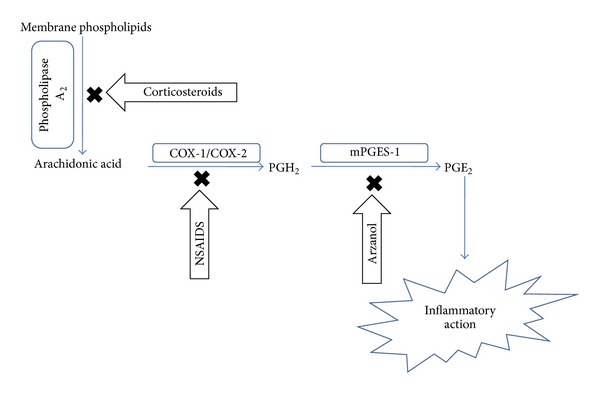
Site of action of arzanol and other anti-inflammatory agents.

Figure 6 reflects the site of action of arzanol; it inhibits the mPGES-1 enzyme which plays a important role in conversion of PGH_2_ to PGE_2_. Formation of PGE_2_ in intact human monocytes was determined to evaluate mechanistic activity of arzanol by Bauer et al. [16]. The results showed arzanol could inhibit PGE_2_ formation either interference with COX-2 expression or mPGES-1. The mPGES-1 inhibitory activity of arzanol was evaluated by preparing crude mPGES-1 in microsomes of IL-1*β*-stimulated A549 cells. Arzanol concentration-dependently inhibited PGE_2_ formation having superior over the mPGES-1 inhibitor MK-886 which was used as control. Despite, heat inactivation of microsomal preparations A549 cells for 15 min at 65°C, the nonenzymatic PGE_2_ formation was not affected by arzanol. It clearly suggests that arzanol is a specific mPGES-1 inhibitor. Arzanol not only is mPGES-1 inhibitor but also proved as inhibitor of other proinflammatory mediators TXB_2_ and leukotrienes by inhibiting 5-LOX and COX-1 enzymes.

*In vivo* model of acute inflammation for evaluation of arzanol was carried out through carrageenan-induced pleurisy in rats. Arzanol (3.6 mg/kg, i.p.) treatment on carrageenan injected in plural cavity of rats significantly reduces exudate formation (59%), cell infiltration (48%), and inhibition of PGE_2_ (47%). However, levels of LTB_4_ and 6-keto PGF_1*α*_ attenuate to only 31% and 27%, respectively. In conclusion, decreased PGE_2_ level may contribute to the inhibition of mPGES-1 by arzanol. Hence,* in vivo* and *in vitro* studies reveal that arzanol is novel eicosanoid inhibitor that importantly acts on PGE_2_ synthesis [16].

 Previously, arzanol proved as a potent NF*κ*B inhibitor [15]. NF*κ*B is involved in activation of large number of genes in response to infections, inflammation, and other stressful situations requiring rapid reprogramming of gene expression [33]. Mechanism of action of arzanol on NF*κ*B inhibition is explained in later part of this paper.

## 5. Other Pharmacological Role

### 5.1. Anti-HIV Activity

 Overwhelming immune activation is the main feature of progression of HIV infection. This immune activation manifested by increased B cell activation, T cell turnover, proinflammatory cytokines, and chemokines [34]. Immune activation leads to generation of activated T cell targets for the virus itself, further viral replication process, and host cell transcription and proliferation. Once new CD4+ T cells are locally activated and infected, they in turn infect other activated cells, amplifying the initial events [35]. Increasing evidence for cytotoxic T lymphocytes (CTL) in the host defence indicates a protective role against human immunodeficiency virus (HIV) whereas their decline is usually coincident with diseased progression [36].

**Figure 7:**
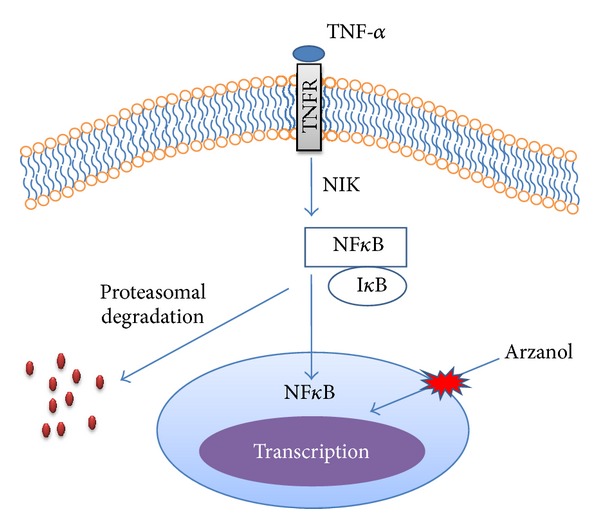
TNF-*α* mediated NF*κ*B signal transduction pathway. TNF-*α* bind to TNFR (TNF-*α* receptor) lead to activation of NF-*κ*B inducing kinase (NIK) which phosphorylates and activates NF*κ*B and I*κ*B (inhibitors of NF*κ*B) complex. IKK then phosphorylates I*κ*B, which leads to its ubiquitination and degradation by the proteasome. NF-*κ*B then enters the nucleus and acts on target genes. Arzanol inhibit this NF*κ*B activation involved in signal transduction pathway. (Adapted and modified from Appendino et al. [15].)

 NF-*κ*B is one of the key regulators of genes involved in the immune/apoptotic response and is implicated in the regulation of several factors viz. cytokines, chemokines, adhesion molecules etc. Arzanol acts as an anti-HIV-1 agent, by inhibiting NF*κ*B activation pathway (Figure 7) [15, 37, 38]. TNF-*α* induced NF*κ*B activation pathway mediates HIV-1-LTR transactivation in a T cell line. This activated pathway inhibited by arzanol was measured using luciferase enzyme level. Results showed that arzanol inhibited luciferase activity in concentration-dependent manner. The overall effect of arzanol shows it has anti-HIV activity by inhibiting HIV-1 replication in T cells. Plant derived phloroglucinols and synthetically derived dimeric phloroglucinols are also being proved for their anti-HIV activity [39].

### 5.2. Antioxidant Activity

 Oxidation is a process of transfer of electron molecule to an oxidizing substance. Oxidative stress develops due to generation of reactive oxygen species, namely, hydroxyl radicals (OH^∙^) and superoxide anions (O_2_^∙−^, OOH^∙^) [40]. These radicals are scavenged by antioxidants which play an important role in the treatment of diabetes mellitus, inflammatory diseases, atherosclerosis, and carcinogenesis [41].

Rosa et al. [17] investigated antioxidant activity on linoleic acid assay, cholesterol assay, and cell culture method. Linoleic acid autoxidation mediated by FeCl_3_, EDTA causes generation of linoleic acid peroxyl radicals (LOO^∙^). Results show arzanol inhibits this oxidation process completely at 5 nmol while 90% and 45% protection occur at 2.5 and 1 nmol concentration, respectively. Cholesterol degradation and formation 7-keto and 7*β*-OH derivatives were significantly inhibited by arzanol (10 nmol) at 1 and 2 h. Oxidative stress in VERO cell line was induced by TBH (*tert*-butyl hydroperoxide) and arzanol showed significant 40% reduction in production of malondialdehyde (MDA). These investigators also proved arzanol is noncytotoxic up to 40 *μ*M *in vitro* tested in VERO cell culture models [17]. 

## 6. Conclusion and Future Perspectives

Arzanol, prenylated heterodimeric phloroglucinyl pyrone, isolated from *H. italicum* subsp. microphyllum. have major anti-inflammatory, antiviral (anti-HIV), and antioxidant activities. Arzanol inhibits NF*κ*B activation, HIV replication in T cells, releases of proinflammatory mediators like IL-1*β*, IL-6, IL-8, and TNF-*α*, and biosynthesis of PGE_2_ by potentially inhibiting mPGES-1 enzyme. Arzanol was widely studied for its pharmacodynamic profile while pharmacokinetic profile still needs to be established. In future, diversity of mechanism of actions of arzanol may be useful in treatment of cancer.

 Although, an extensive amount of research work has been done on anti-inflammatory compounds to date, mPGES inhibitors like arzanol are still unknown. Only a few species from genus *Helichrysum* have been investigated [42]. Consequently, a broad field of future research remains possible in which the isolation of new active principles (mPGES-1 inhibitors) from the genus *Helichrysum *would be of great scientific merit.

## Figures and Tables

**Figure 3:**
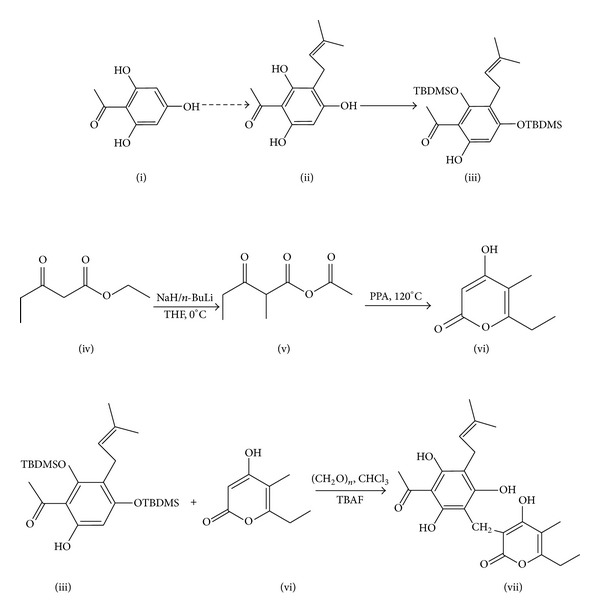
Synthesis of arzanol. (Adapted from Minassi et al. [20, 21].)

## Abbreviations

COX: Cyclooxygenase
CTL: Cytotoxic T lymphocytes
HIV: Human immunodeficiency virus
IKK: I*κ*B kinase
IL: Interleukins
LOX: Lipoxygenase
MDA: Malondialdehyde
mPGES: Microsomal PGE_2_ synthase
NF*κ*B: Nuclear factor kappa B
NIK: NF-*κ*B inducing kinase
OVLT: Organum vasculosum lamina terminalis
PAF: Platelet-activating factor
PGD_2_: Prostaglandin D_2_
PGDS: Prostaglandin D synthase
PGE_2_: Prostaglandin E_2_
PGES: Prostaglandin E synthase
PGF_2*α*_: Prostaglandin F_2*α*_
PGFS: Prostaglandin F synthase
PGG_2_: Prostaglandin G_2_
PGH_2_: Prostaglandin E_2_
PGI_2_: Prostaglandin I_2_
PGIS: Prostaglandin I synthase
PLA_2_: Phospholipase A_2_
POA: Preoptic area
TBAF: Tetrabutylammonium fluoride
TBDMSCl: *tert*-Butyldimethylsilyl chloride
TBH: *tert*-Butyl hydroperoxide
TNF-*α*: Tumour necrosis factor-*α*
TPA: Tissue plasminogen activator
TPP: Tetraphenylporphyrin
TXA_2_: Thromboxane A_2_
TXS: Thromboxane synthase.
